# Pre- and post-testing counseling considerations for the provision of expanded carrier screening: exploration of European geneticists’ views

**DOI:** 10.1186/s12910-017-0206-9

**Published:** 2017-08-01

**Authors:** Sandra Janssens, Davit Chokoshvili, Danya F. Vears, Anne De Paepe, Pascal Borry

**Affiliations:** 1 Centre for Medical Genetics Ghent, University Hospital Ghent, De Pintelaan 185, 9000 Ghent, Belgium; 2 Centre for Biomedical Ethics and Law, Department of Public Health and Primary Care, KU Leuven - University of Leuven, Leuven, Belgium; 3 Leuven Institute for Human Genomics and Society, KU Leuven - University of Leuven, Leuven, Belgium

**Keywords:** Expanded carrier screening, Pre-test counseling, Post-test counseling, Interviews

## Abstract

**Background:**

Carrier screening is generally performed with the aim of identifying healthy couples at risk of having a child affected with a monogenic disorder to provide them with reproductive options. Expanded carrier screening (ECS), which provides the opportunity for multiple conditions to be screened in one test, offers a more cost-effective and comprehensive option than screening for single disorders. However, implementation of ECS at a population level would have implications for genetic counseling practice.

**Methods:**

We conducted semi-structured interviews with sixteen European clinical and molecular geneticists with expertise in carrier screening to explore their views on the implementation of ECS in the clinical setting.

**Results:**

Using inductive content analysis, we identified content categories relevant to the pre- and post-test settings. Participants believed ECS would ideally be targeted at couples before pregnancy. There was some disagreement regarding the acceptability of performing ECS in individuals, with several participants actively opposing individual-based screening. In addition, participants discussed the importance of ensuring informed and voluntary participation in ECS, recommending measures to minimize external pressure on prospective parents to undergo testing. A need for adequate counseling to foster informed, autonomous reproductive decision-making and provide support for couples found to be at risk was emphasized.

**Conclusions:**

Practical challenges in optimizing pre-test education and post-test counseling should not be underestimated and they should be carefully addressed before implementing ECS in the clinical setting.

## Background

Carrier screening aims to identify couples at risk of having a child affected with a monogenic (autosomal or X-lined) recessive disorder. In autosomal recessive disorders, both members of an at-risk couple are usually unaffected carriers of a single faulty copy of the gene associated with the same disorder, while in X-linked disorders, only the female partner is a carrier. In both cases, such couples (henceforth referred to as carrier couples) run a 25% chance of conceiving an affected child in each pregnancy. Since the majority of carrier couples have no family history of the disorder, they are often unaware of their reproductive risks [1]. Thus, identification of carrier couples via carrier screening has the potential to enhance their reproductive autonomy, allowing them to make informed decisions. Carrier couples have the option to utilize preimplantation genetic diagnosis or prenatal diagnosis to avoid having an affected child [2], or can conceive naturally, prepare for the birth of a potentially affected child and, in some cases, initiate treatment in the newborn period [3].

Traditionally, due to technical and economic constraints, carrier screening had been performed for single disorders such as cystic fibrosis (CF), spinal muscular atrophy (SMA), or fragile X, and included a limited set of pathogenic mutations. Expanded carrier screening (ECS) represents an evolution from single- to multi-disease screening panels, driven by continuous advances in genomic technologies and improved understanding of the genetics of monogenic disorders [4]. Screening for upwards of a hundred recessive disorders in a single test, ECS has the potential to detect more carrier couples at risk of having an affected child in the general population. With the decrease in costs and improvements in carrier detection rates, ECS is rapidly becoming an integral part of reproductive healthcare [5, 6]. In particular, in the US, more than 200,000 ECS tests are performed in prospective parents annually, primarily through commercial genetic testing providers [7]. In Europe, adoption of ECS has been relatively slow, with testing being largely limited to high-risk ethnic populations and patients undergoing artificial reproduction [8, 9]. However, recent studies have shown that members of the general public in Europe are interested in ECS [10, 11] and the demand for and uptake of ECS tests is likely to increase in many European countries in the near future.

As with all new medical technologies, adoption of ECS comes with its own set of practical and ethical challenges. In particular, issues relating to devising optimal screening strategies, provision of genetic counseling, and developing the best clinical practices will need to be addressed in the near future. In the US, several studies have investigated geneticists and other healthcare providers’ opinions on ECS [5, 12–14] and some professional organizations have developed recommendations concerning its use [15, 16]. However, in Europe, discussion on ECS has only recently gained traction, with the first professional document addressing ECS published in 2016 [17]. In this paper, we report European geneticists’ attitudes towards ECS, focusing on their concrete suggestions and recommendations for the use of ECS in the clinical setting.

## Methods

This study utilized semi-structured interviews to explore the views of European geneticists on various aspects of ECS. The interview guide used in the study consisted of open-ended questions addressing various issues related to ECS, with a particular emphasis on the challenges to implementing ECS into clinical practice. Our goal was to perform expert interviews with geneticists possessing extensive research, clinical and/or counselling experience in carrier screening [18]. To this end, we employed purposive sampling to identify potential participants, taking into consideration their authorship of relevant academic publications, conference abstracts and policy papers. As the recruitment continued concurrently with the interviews, we additionally asked our participants to nominate their colleagues who they believed were qualified to discuss ECS-related issues (snowball sampling).

Potential participants were approached by email and those who agreed to participate were interviewed during the period April to August 2014, either in person or via Skype. Informed consent was obtained from all participants included in the study. The questions addressed in this paper were part of a study exploring ECS more broadly, including recommendations for the composition of ECS panels and the desirability of population-wide ECS [19, 20].

Inductive content analysis was used to identify common content categories from the interviews [21–23]. The data resulting from the interviews were initially coded into broad categories using the qualitative data management software QSR Nvivo. This initial phase of coding was performed by DC for all the interview transcripts and cross-validated by DV. In the subsequent phase, sections of the data within the broad categories were compared by all members of the research team and specific content categories were proposed in group discussions. Decisions on the specific categories were reached by consensus.

The study was approved by the institutional ethics committee of the University Hospital Ghent.

## Results

We interviewed sixteen geneticists based in eight European countries (member states of the European Economic Area). This group comprised thirteen clinical geneticists (CG), two molecular geneticists (MG), and one geneticist with professional experience in both molecular and clinical genetics (CMG). Most participants (12/16) had more than 20 years of professional clinical or diagnostic experience at the time of the study and all held an academic affiliation.

In total, participants discussed four main concepts relating to the provision of ECS: *(1) strategies for offering screening*, *(2) ensuring informed decision-making, (3) communication of individual carrier status, and (4) counseling of carrier couples.* We have organized these into two broad categories, depending on whether they relate to pre- or post-test setting (Table 1). These categories, along with illustrative quotes, are described below.

**Table 1:** An overview of content categories discussed during the interviews

Category 1. Pre-test considerations	
Subcategory 1.1 Strategies for providing population-wide ECS • *Timing of screening* • *Issues with screening of individuals*	
Subcategory 1.2 Ensuring informed decision-making • *Providing neutral information about ECS* • *Nondirective offers of testing* • *Informed decisions about disorders to be screened*	
Category 2. Post-test considerations	
Subcategory 2.1 Communication of individual carrier status	
Subcategory 2.2 Counseling of carrier couples	

### Category 1. Pre-test considerations

#### Subcategory 1.1. Strategies for providing population-wide ECS

Participants discussed optimal strategies for offering ECS to prospective parents, focusing on two aspects: the *timing of screening* and whether *screening individuals* was appropriate, compared to only offering screening to couples*.*

##### Timing of screening

All participants agreed that ECS should ideally be offered to couples in the preconception period, when reproductive partners have already made their plans about having children.

*“[T]he most proper group would probably be couples which are planning, or would be planning to have a baby. Not being pregnant yet, and not being too far away from the pregnancy.” (Interview 1, CG)*

Nevertheless, most participants acknowledged the importance of also allowing people access to ECS during pregnancy. Several participants noted that the lack of awareness of carrier screening among non-pregnant couples, and the possibility of unplanned pregnancies, suggests couples may only find out about ECS in the prenatal period.

*“When you have a pregnancy it is too late. But unfortunately there is going to be a lot of cases like that, because this is what you see now.” (Interview 10, CG)*

##### Issues with screening of individuals

All participants agreed that screening both partners was optimal, and supported a couple-based approach to carrier screening. Participants also discussed the possibility of screening individual prospective parents seeking carrier screening without a reproductive partner. In general, participants viewed screening of individuals in this situation as more problematic, articulating several reasons against this approach. However, there was no agreement regarding rejection of individual-based screening: while some argued that ECS should be provided exclusively to couples, several participants believed that ECS could also be offered to individuals. Most participants who were against screening of individuals, drew attention to the lack of clinical utility in this approach.

*“[The] goal of the preconception screening is to find carrier couples. It’s not interesting to find individual carriers because this doesn’t have any consequences. Any medical consequences.” (Interview 5, CG)*

For another participant, screening of individuals was morally problematic due to the possibility that this approach could motivate some prospective parents to select their future reproductive partner based on genetic compatibility.

*“No, I’m not in favor of that [screening individuals while they are single]. I find it a strange thing. [This may lead to] a whole thing of matching people, you know, all over the world.” (Interview 12, CG)*

According to another participant, undergoing carrier screening is a decision that has to be made by both members of the couple and must be mutually agreed on.

*“I do think that this is something that they have to do together. Not just because for us it’s more informative to have both partner[s]. But this is also a process that must be done together as a couple. So* ***we*** *want a baby,* ***we*** *are getting tested in order to know if* ***we*** *are carriers … So it’s something* ***we*** *are doing together, not just the woman goes and gets tested.” (Interview 7, MG; emphases by the participant)*

On the other hand, those participants who favored providing ECS to individuals emphasized the importance of the carrier status information for family members.

*“[T]here are family implications of these results. So it’s hard to say it’s just something which is important as a couple, because I mean, it can be important for the family members as well.” (Interview 13, CG)*

Another participant mentioned that there may be value in performing individual-based carrier screening as it reduces uncertainty in prospective parents who are worried that they may be carriers based on their family history. However, they also noted that screening of individuals for autosomal recessive disorders does not carry public health benefits and thus should only be performed in the commercial setting.

*“[You] have to offer it to individuals … [because] in the real world, somebody who has a [relative] with [a recessive disorder], is going to ask for it. So, if they want to pay for it privately, go ahead and have [it].” (Interview 9, CG)*

#### Subcategory 1.2 Ensuring informed decision-making

All participants discussed the importance of couples (or individuals) making informed decisions about undergoing ECS. To achieve this, participants identified three necessary conditions: *providing neutral information about ECS, nondirective offers of testing,* and *informed decisions about disorders to be screened.*

##### Providing neutral information about ECS

Participants suggested that in order to facilitate access to screening in the preconception period, efforts should be made to inform the public about ECS. However, several participants were concerned that prospective parents may then feel obliged to take the test.

*“If you are going to offer it, there must be some public campaign, saying that it’s there. But … if you are making a very big campaign, then people would think ‘oh, I should do that, because otherwise I’m stupid and it’s my own choice to have a baby with a handicap’. And that’s not the message I want to bring across.” (Interview 3, CG)*

Some participants were worried that concerns over the health of one’s future child could make prospective parents susceptible to pressure from ECS providers, particularly if it is offered by direct-to-consumer genetic testing companies. According to some, these companies tend to downplay the limitations of their products and overstate the potential benefits of carrier screening, which *“might lead to unrealistic expectations” (Interview 16, CG).* Consequently, the majority of interviewees expressed their strong disapproval of direct-to-consumer ECS products.

*“When you look at the website of [name of a company] for example, the text on [it] is just awful. It’s like: ‘who doesn’t want to prevent the birth of a child with a genetic disease?’ And ‘it’s your responsibility’. And it’s a very coercive way of informing the people.” (Interview 2, CG)*

Our participants argued that pre-test information provided to prospective parents should clearly emphasize the limitations of ECS, including that ECS may identify mutations with limited clinical significance but also fail to identify some carriers.

*“[E]ither you have to tell them: ‘I may find stuff that I’ll not be able to explain to you’ or you have to tell them: ‘I will only look at the known stuff [and] I’m going to give you a … more informed answer, but this is going to cover slightly less. So you’ll still have a chance of having a child with a recessive disorder.” (Interview 10, CG)*

##### Nondirective offers of testing

According to the participants, as many prospective parents in the general public are likely to be unaware of the option of carrier screening, discussions about ECS would generally need to be initiated by a healthcare provider. However, participants cautioned that in order to allow prospective parents sufficient time to weigh the advantages and disadvantages of ECS and make an informed decision consistent with their values, the actual offer of screening should not take place immediately.

*“I wouldn’t want … [testing] to be very closely linked in time to the information-offering. Because if it forces people to make choices quickly, then really what they need is time and space to think about that.” (Interview 14, CG)*

To ensure the offer of ECS is non-directive, several participants suggested the main role of healthcare professionals in the pre-test setting should be to explain the basics of ECS to their patients and provide them with appropriate educational resources to peruse in their own time.

*“I think that you should explain, but very briefly, the mode of transmission. You should explain that the father, the mother, they are healthy but they are carriers of the disease. … [For the rest,] you should also have for some people some leaflets … [also, you should tell them]: ‘you can go on this site, and be careful about the other site’ … so when they are at home, if they wish, they [can access] it.” (Interview 15, CG)*

In general, our participants supported the use of audiovisual aids, such as leaflets, websites, and brochures, as an alternative to detailed pre-test counseling. This was recommended to avoid overloading prospective parents with too much information during a consultation, reduce the workload for healthcare providers, and ensure that all prospective parents receive the same, high-quality information.

*“I could well imagine a system where you have a written leaflet and a very well, carefully constructed YouTube 5-10 minute educational video clip that people will be asked to access before testing. … It’s [important] that people have leaflets, something web-based educational, information-giving resource.” (Interview 14, CG)*

##### Informed decisions about disorders to be screened

Participants were concerned that due to the large number of disorders that can be included on ECS panels, prospective parents would have difficulty understanding the vast amount of information necessary to make informed decisions regarding the disorders for which they would want to be screened.

*“[I]f you make a test where there are as well diseases [leading to] babies dying in the first days of life and diseases where they might become blind at the age of twenty, that’s a totally different kind of decision for the people … [I]t’s all very different diseases, very hard to make an informed choice.” (Interview 3, CG)*

Following this logic, some argued that asking potential parents to consent to screening for specific disorders would be impractical, and that a more generic consent would be preferable.

*“You cannot ask a parent ‘do you want screening for this disease and not for that disease?’ … You have to give permission for all diseases and when the test is positive for one of the diseases, then you get detailed information.” (Interview 5, CG)*

Despite the practical benefits of employing a generic consent for ECS, some also pointed out that this approach fails to recognize differences among various disorders, which they considered a weakness of this approach. As a potential solution, a tiered approach to consent was proposed, where diseases could be grouped into categories based on common characteristics. This would simplify the information for potential parents to consider when making decisions about screening.

*“You have to cluster [disorders] … [For example:] There are diseases that are lethal in the first few years of life and … this is for severe mental handicap… [In this way,] people can make a decision: ‘I want to know whether my baby has a risk of a lethal disease or mental retardation.” (Interview 3, CG)*

### Category 2. Post-test considerations

When discussing the post-test setting, our participants focused on issues such as communication of results, genetic counseling, education and psychological support of carrier couples.

#### Subcategory 2.1 Communication of individual carrier status

Participants who supported screening individuals believed that it would be sufficient to communicate these results either in written form or verbally by a non-genetics healthcare provider. According to these participants, reports should emphasize the meaning of the results.

*“[It should be stated] in the report after that it’s always a couple issue. So, you are a carrier, it’s not a problem, everybody is a carrier of something … [s]hould [your partner] be a carrier of the same … thing as you, then there would be a risk.” (Interview 6, CMG)*

Most participants agreed that in the case of couple-based screening, both members of the couple should have access to their individual test results. Interestingly, some participants who opposed screening individuals were comfortable providing individual test results to both partners in couple screening.

“*I believe that [when] you give them the answer, then you should tell them whatever [each of them] carries. You’ll tell them: ‘look, you are carrying that, you’re carrying that, there is no problem.’ … [M]y feeling is that if you test the people and you find something, at least they must have an option to know.” (Interview 10, CG)*

Another participant argued that individual carrier status can be important in future relationships, expressing support for individual-based reporting of test results.

*“[People may] change their partners. It’s a quite common practice now. So … I think we have to [inform individual carriers].” (Interview 8, CG)*

While participants generally felt uneasy about not disclosing test results to couples, there was also some discomfort in disclosing individual carrier results because, as this participant stated, “carriership in one person of the couple and not in the other is also unwanted information, because that’s not important if you stay with the same partner” (Interview 5, CG). In addition, one participant displayed reluctance towards communicating individual results to the members of a screened couple, stating that “technical problems of deciding in one person if what you find is a pathogenic mutation … are too [great]” (Interview 4, CG). In particular, they argued that outside a small set of highly penetrant pathogenic mutations, identifying persons as carriers of autosomal recessive disorders based on their individual test results would be misleading. According to this participant, since the reproductive risk and the expected phenotype of the affected child would depend on the joint genotypes of both partners, individual test results should generally not be communicated to couples undergoing ECS.

Finally, one participant was especially vocal in their opposition to the disclosure of individual results, particularly in the context of a public health program which aims to identify carrier couples.

*“I think if you are trying to set up a public health exercise and to identify couples at increased risk of a child with a severe recessive condition, then you should stick to that [goal] and not communicate things that are not [relevant] to that. … ‘[You should] make it very clear that even if they say ‘I want my individual results’, that’s not something … that you feel is justified in your public health exercise. … If your aim is something else altogether, then you might say differently.” (Interview 16, CG)*

#### Subcategory 2.2 Counseling of carrier couples

All participants agreed that face-to-face genetic counseling is indicated for couples where screening identifies that both members carry a pathogenic mutation associated with the same autosomal recessive disorder, or where the female is a carrier of an X-linked disorder.

*“[E]veryone who has a really high risk, carrier couples, need genetic counseling. They are not well-off with only a website or something.” (Interview 4, CG)*

There was a consensus among participants that the goal of post-test genetic counseling is to assist carrier couples in making informed reproductive decisions. Participants believed that counseling should include an explanation of the genetic condition and discussion of their reproductive options. However, the difficulty associated with explaining the clinical aspects of a disorder, given the couple may have no preexisting experience with the condition, was noted.

*“You are giving … the carrier couple information about a condition of which they don’t have a first-hand experience [with]. So, that imposes anxieties and difficult decision processes on them. … [Because you can’t] easily give an understanding of life with the condition and its severity and impact on personal and family life.” (Interview 14, CG)*

To address this, several participants suggested involving a medical professional with expertise in the disease. They felt that post-test counseling and follow-up should be provided neutrally and non-directively to allow prospective parents to make uncoerced reproductive decisions that are in line with their values.

*“[I]f parents say, ‘ok’ – I have had already parents even if they had a child with CF and say – ‘ok, I take my chance, and I will have a child without any prenatal diagnosis’, I respect completely, really. It’s my opinion. I agree with all the choices of the parents.” (Interview 15, CG)*

## Discussion

In this study, we have reported the views of European clinical and molecular geneticists regarding the pre- and post-test considerations required to implement population-wide expanded carrier screening in an ethically sound way.

All the participants in our study believed that carrier screening would ideally be offered to prospective parents in the preconception period, which would maximize reproductive autonomy for carrier couples by providing both the greatest range of reproductive options and sufficient time for decision-making. However, due to the possibility of unplanned pregnancies, and limited awareness of carrier screening among couples in the preconception period, our participants believed carrier screening should also be made accessible to pregnant couples, as suggested by others [3, 17, 24–26].

We noted disagreement among participants regarding whether it was appropriate to offer screening to individuals, with some arguing that ECS should be performed exclusively in couples. Although professional genetic organizations have endorsed both approaches [16, 17], some of our participants identified important issues associated with performing carrier screening in individuals. First, in autosomal recessive conditions, learning one’s individual carrier status is insufficient to estimate reproductive risks when a partner is not available for screening. Should one’s (future) partner decline participation, tensions may arise within the relationship and the individual who had undergone ECS would be left with incomplete information. Unavailability of the reproductive partner for testing was also identified as an important barrier to individual carrier screening in a recent focus group study with women who had undergone a genomic carrier test [27]. Another potential advantage of couple-based screening is the possibility to design panels to better meet the objectives of the screening program. If the program aims to identify only the couples at risk of having children affected with a severe phenotype, a couple-based approach may achieve this by selectively excluding carrier couples with milder genotypes. For example, in mutations where more severe phenotypes are associated with a compound heterozygous state while homozygosity typically leads to a mild phenotype (such as R117H in cystic fibrosis or N370S in Gaucher disease) [28–30], the screening program may seek to identify couples at risk of having a compound heterozygous child, while avoiding identification of couples who may have a homozygous child. This would only be possible if screening were to be performed exclusively in couples. Furthermore, reporting of results would also need to be couple-based, individual results would be withheld, and, only couples at risk of having children with a severe phenotype would be informed of their reproductive risks, whereas all others would receive negative results [31]. However, this strategy is controversial as some results are not communicated [32, 33], the opportunity to initiate cascade screening among family members of individual carriers is foregone [25, 33], and individual carriers cannot use their test results in subsequent relationships [25]. Limiting carrier screening to couples would only be reasonable in the case of autosomal recessive disorders, since in X-linked recessive conditions, reproductive risks can be accurately estimated even in the absence of the male reproductive partner. Identifying some of these challenges, our participants generally agreed that both partners should be entitled to receive their individual results when they are screened as a couple. Given both individual- and couple-based approaches to screening have some disadvantages, preferences between these two strategies may vary across cultures and it may therefore be appropriate to make the decision about whether to screen individuals on a case-by-case basis, rather than through the development of over-arching policies.

Participants also suggested prospective parents should be provided with objective information to minimize the possibility of coercion, preferably through appropriately designed patient decision aids, such as videos, interactive multimedia, and paper-based materials. This approach would, as has been suggested by other authors, reduce the burden on healthcare providers who may lack both the time and the necessary expertise to educate their patients about ECS [34–36] and allow users to access and process the information at their own pace [37]. Furthermore, it also acknowledges that prospective parents are likely to require varying levels of information prior to testing with some of them not desiring a detailed understanding of ECS [38]. Finally, receiving a carrier screening offer from a medical professional, regardless of how nondirective, may be misconstrued as an implicit recommendation to take the test [25, 39], potentially influencing the decision regarding screening. Due to the sensitive nature of reproductive decisions, our participants highlighted the necessity of ensuring that undergoing ECS is a fully informed and voluntary decision that corresponds with the couple’s personal values. To this end, our participants recommended that although in many cases a healthcare provider would initiate discussions about ECS, this should be limited to a brief explanation of ECS and provision of educational materials. Additionally, several participants emphasized that requiring a repeat-visit for testing, instead of performing ECS in the same-visit, would ensure that future parents are making an active decision and are motivated to undergo testing. This approach to carrier screening has been described previously, with authors arguing that requiring a repeat-visit would maximize voluntary participation [40, 41]. However, the presence of a barrier in accessing carrier screening, such as the need for second visit, is likely to result in a lower uptake of the test, as seen in studies of cystic fibrosis screening programs [42]. It is important that the providers of carrier screening consider the potential trade-off between informed and voluntary participation on the one hand and uptake on the other.

Although utilizing educational materials has the potential to increase informed decision-making, this goal can only be accomplished if the information is presented in an objective and neutral way. Our participants held concerns that this may be more challenging if carrier screening is offered by direct-to-consumer genetic testing companies who employ audiovisual aids primarily for advertising purposes [35, 43]. Since many prospective parents are highly motivated to do everything possible to ensure the health of their offspring [27], they may be susceptible to persuasion by advertisements which suggest ECS as a guarantee to a healthy baby. Thus, it is important that pre-test educational tools are developed with the aim of objectively informing prospective parents about the benefits and disadvantages of ECS. To this end, professional medical and genetic organizations, rather than commercial providers of ECS, may be best-placed to produce educational tools that convey objective information, including the limitations of ECS, to prospective parents [16, 17].

In accord with discussions about the infeasibility of disease-specific informed consent in the context of ECS that have existed since the 1990s [44], our participants believed it is impractical to solicit informed consent for every disorder on a panel when screening for large numbers of disorders. Generic consent, which would broadly describe the nature of the test, instead of focusing on the medical aspects of individual conditions, has been proposed as a viable alternative for ECS with considerable advantages [15]. However, our participants suggested that in order to enhance reproductive autonomy, prospective parents should be able to choose which disorders they would like to be screened for, rather than being locked into an all-or-nothing offer for a panel of diverse disorders [45]. To address this, some commentators have proposed clustering disorders into different categories or tiers based on characteristics such as severity or age of onset, and allowing patients to make choices between these categories [38, 46]. A tiered approach to consent was supported by some of our participants, who viewed it as a middle ground solution between disease-specific and generic consents. However, it is unclear whether clustering disorders into multiple categories is helpful for prospective parents to make informed discriminatory choices among the categories. The utility of a tiered consent is currently being evaluated and, if shown to be beneficial, may gain further support within the professional genetic community [47].

Our participants believed that face-to-face post-test genetic counseling discussing the clinical characteristics of the disorder and their available reproductive options should be provided to all carrier couples identified by ECS. The medical information provided to carrier couples should be comprehensive and up-to-date, including an overview of the latest and emerging therapeutic interventions [48]. To this end, our participants felt carrier couples would benefit from an additional consultation with a medical professional with clinical expertise in the disorder and also the opportunity to interact with affected individuals, or their family members. Some patient organizations have also recommended the availability of patients or their family members for a consultation, to help at-risk future parents to gauge the burden of the disease and make informed reproductive decisions [49]. Importantly, while this may be possible in relatively common disorders, such as CF, couples at risk of having a child with a rare disorder may not have the same opportunities. Therefore, in many cases carrier couples will be reliant on genetic counselors to help them contemplate the potential impact of having an affected child. Therefore it is crucial that genetic counselors are prepared to provide information and support about all the disorders on the ECS panel.

The findings of our study are in some respects comparable to those presented by Cho et al. and McGowan et al., based on focus group discussions involving geneticists from the US. In both studies, geneticists emphasized the importance of pre- and post-test information and counseling aimed at facilitating informed decision making among prospective parents [12, 13]. However, we observed strong disagreement among our participants regarding the acceptability of screening individuals, which was not described is the US-based study. In addition, according to some participants in our study, in order to ensure that only motivated prospective parents take the test, it may be appropriate to add certain barriers to test access, such as requiring a repeat-visit. This differs from the findings of McGowan et al., where participants believed that most healthcare providers will be compelled to routinely offer ECS in order to avoid liabilities should an affected child be born and did not discuss strategies aimed at maximizing informed decision-making [13]. The possibility of holding healthcare providers accountable due to their failure to offer ECS was conspicuously absent in our interviews, which may be explained by differences in legal frameworks governing the practice of reproductive medicine.

## Limitations

Our study purposively sampled geneticists with expertise in carrier screening for recessive disorders. Therefore, while the study provides valuable insights into the key ethical and practical challenges associated with the implementation of ECS, their opinions are not necessarily generalizable to those of geneticists in Europe more broadly. Future studies including participants without expert knowledge in carrier screening would be a valuable contribution to the literature. In the context of population-wide screening the task to initiate discussions about ECS with prospective parents is likely to fall on healthcare providers, such as obstetrician-gynecologists and general practitioners, rather than geneticists. However, some of these non-geneticist healthcare professionals may not be well-prepared to provide adequate guidance and genetic counseling to their patients regarding ECS. Therefore, exploring attitudes of non-genetics professionals should also be undertaken in order to gain a more comprehensive understanding of the practical challenges they foresee with the implementation of ECS in the public health setting. Finally, it should be noted that even though most ECS tests are currently offered through commercial genetic testing companies, the views of commercial providers regarding ECS remain largely unexplored. Thus, it is important that future empirical studies focus on the views of commercial providers, such as direct-to-consumer genetic testing companies.

## Conclusions

We identified that our participants placed high importance on ECS being offered preconceptionally and in the setting of couple-based screening. This highlights a need for both adequate education and counseling services, but also underscores the importance of making efforts to facilitate access to ECS before pregnancy. To ensure that prospective parents understand the complex information pertaining to ECS, pre-test information can be provided through patient-centered education aids, particularly videos and computer-based interactive tools. In the post-test setting, in-person genetic counseling is indicated in couples found to be at risk of conceiving an affected child. However, practical challenges in optimizing pre-test education and post-test counseling should not be underestimated and they should be carefully addressed before implementing ECS in the clinical setting.

Our study has also provided valuable insights into the important differences between individual and couple-based strategies in carrier screening. While the superiority of either of the approaches cannot be determined without taking into consideration the local context, it is important that decision-makers acknowledge substantial differences between the two approaches and give due consideration to identifying the optimal screening and reporting strategies. Future studies need to focus on the preferences between the two approaches among different stakeholders, such as healthcare providers offering ECS and couples undergoing carrier screening.

## Abbreviations

CF: Cystic fibrosis
CG: Clinical geneticist
CMG: Clinical and molecular geneticist
ECS: Expanded carrier screening
MG: Molecular geneticist
SMA: Spinal muscular atrophy
